# Vaccination with *L. infantum chagasi* Nucleosomal Histones Confers Protection against New World Cutaneous Leishmaniasis Caused by *Leishmania braziliensis*

**DOI:** 10.1371/journal.pone.0052296

**Published:** 2012-12-20

**Authors:** Marcia W. Carneiro, Diego M. Santos, Kiyoshi F. Fukutani, Jorge Clarencio, Jose Carlos Miranda, Claudia Brodskyn, Aldina Barral, Manoel Barral-Netto, Manuel Soto, Camila I. de Oliveira

**Affiliations:** 1 Centro de Pesquisas Gonçalo Moniz, The Oswaldo Cruz Foundation, Salvador, Brazil; 2 Instituto de Investigação em Imunologia, Salvador, Brazil; 3 Centro de Biología Molecular Severo Ochoa, Consejo Superior de Investigaciones Cientificas, Departamento de Biologia Molecular, Universidad Autonoma de Madrid, Madrid, Spain; University of São Paulo, Brazil

## Abstract

**Background:**

Nucleosomal histones are intracellular proteins that are highly conserved among *Leishmania* species. After parasite destruction or spontaneous lysis, exposure to these proteins elicits a strong host immune response. In the present study, we analyzed the protective capability of *Leishmania infantum chagasi* nucleosomal histones against *L. braziliensis* infection using different immunization strategies.

**Methodology/Principal Findings:**

BALB/c mice were immunized with either a plasmid DNA cocktail (DNA) containing four *Leishmania* nucleosomal histones or with the DNA cocktail followed by the corresponding recombinant proteins plus CpG (DNA/Protein). Mice were later challenged with *L. braziliensis,* in the presence of sand fly saliva. Lesion development, parasite load and the cellular immune response were analyzed five weeks after challenge. Immunization with either DNA alone or with DNA/Protein was able to inhibit lesion development. This finding was highlighted by the absence of infected macrophages in tissue sections. Further, parasite load at the infection site and in the draining lymph nodes was also significantly lower in vaccinated animals. This outcome was associated with increased expression of IFN-γ and down regulation of IL-4 at the infection site.

**Conclusion:**

The data presented here demonstrate the potential use of *L. infantum chagasi* nucleosomal histones as targets for the development of vaccines against infection with *L. braziliensis*, as shown by the significant inhibition of disease development following a live challenge.

## Introduction

Leishmaniasis is an infectious disease with significant economic impact in several countries. Over three hundred million people are exposed to the parasites, with 12 million infected worldwide, predominantly in tropical and subtropical countries (World Health Organization page: http://www.who.int/emc/diseases/leish/leisdis1.html). Leishmaniasis can be caused by different species of *Leishmania spp.* protozoans that infect macrophages in the human host. The treatments available for all forms of leishmaniasis are toxic, and drug resistance is on the rise, further increasing the need for vaccine development [1].

Numerous attempts have been made to find a protective antigen against leishmaniasis and several candidates have been tested for this purpose [2], [3], including histones. Histones are structural proteins found in the nucleus, where they play an important role in the organization and function of chromatin. There are five main classes of histones; four of them (H2A, H2B, H3 and H4) form the nucleosomal core unit of chromatin, whereas H1 joins to linker DNA. The percentage of similarity between *Leishmania* nucleosome forming histones and their mammal counterparts ranges from 49% (for the H2B) to 63% (for the H3) [4]. Differences are mainly located in the aminoacid sequences of the nucleosome-exposed tails of the four histones [5]. So far, no cross reactivity was found between *Leishmania* histones and their mammalian counterparts. Antibodies specific for parasite histones, obtained from dogs with visceral leishmaniasis, react against *Leishmania* H2A [6], H3 [7], H2B and H4 [8] but do not recognize mammalian histones. Antibodies in sera from patients with cutaneous or mucocutaneous leishmaniasisis also recognize parasite histone H1 but not the human counterpart [9]. Regarding the T cell immunogenicity of parasite histones, recombinant versions of H2B [10] or H2A [11] induced IFN-γ secretion upon stimulation of PBMCs obtained from cutaneous leishmaniasis patients. The T cell response was specific for parasite histones, since T cell lines derived from cutaneous leishmaniasis patients did not respond to mammalian histones [12].

*Leishmania* histones are recognized by sera from cutaneous leishmaniasis patients [13] and from dogs infected with *Leishmania* [6], [7], [8] and it has been hypothesized that these parasite proteins may trigger an immune response after active destruction or spontaneous cytolysis of *Leishmania* amastigotes [4], [14]. Immunization with the histone H1 was able to confer protection against *L. major* [15], [16] and *L. infantum* [17]. Immunization with H2B was able to confer protection in mice infected with *L. major* [18], as seen by a decrease in parasite load and lesion size. The protective effect against leishmaniasis was also observed upon immunization with the four core histone proteins (H2A, H2B, H3 and H4) [19], [20], [21].

In addition to antigen selection, the immunization strategy is also important for generating protection. Various antigens have been tested and evaluated as DNA and/or recombinant protein vaccine candidates in murine models of leishmaniasis, resulting in various degrees of protection [22]. DNA vaccination has also been tested in heterologous prime-boost vaccination regimes [23], in which the immune system is primed with DNA and boosted with a different formulation of the corresponding antigen. This strategy has been shown to be effective in experimental models of cutaneous leishmaniasis [24], [25], [26].

Based on the protective capacity of the four nucleosomal histones against *L. infantum chagasi* infection, we hypothesized that this antigenic cocktail would confer protection against the development of New World cutaneous leishmaniasis caused by *L. braziliensis,* a species for which there are few published studies regarding vaccine development in comparison with *L. major* [27]. The present study also compared immunization strategies involving plasmid DNA only and plasmid DNA plus recombinant proteins.

## Materials and Methods

### Mice and Ethics Statement

Female BALB/c mice, 6–8 weeks of age, were obtained from CPqGM/FIOCRUZ animal facility where they were maintained under pathogen-free conditions. Animals were randomly distributed into groups of five and each group was subjected to a specific immunization strategy. All animal work was conducted according to the Guidelines for Animal Experimentation of the Colégio Brasileiro de Experimentação Animal and of the Conselho Nacional de Controle de Experimentação Animal. The local Ethics Committee on Animal Care and Utilization (CEUA) approved all procedures involving animals (CEUA – Centro de Pesquisas Gonçalo Muniz/FIOCRUZ - L031/08).

### Preparation of DNA Plasmids and Recombinant Proteins

The recombinant plasmids [21](pcDNA3-LiH2A, pcDNA3-LiH2B, pcDNA3-LiH3 and pcDNA3-LiH4) were prepared using the endotoxin-free Giga-preparation Kit (Qiagen) following the manufacturer’s instructions. The final pellet was resuspended in sterile water and stored at −20°C until use. Expression and purification of the His-tagged recombinant proteins (pQEH2A, pQE-H2B, pQE-H3 and pQE-H4) were performed as previously described [21]. After binding to a Ni-NTA agarose column (Qiagen), recombinant proteins were gradually refolded on the affinity column as described [28]. Recombinant proteins were eluted with 0.3 M imidazole and dialyzed against PBS. Finally, proteins were passed through a polymyxin-agarose column (Sigma) in order to eliminate endotoxins. Residual endotoxin was measured with Quantitative Chromogenic *Limulus* Amebocyte assay (QCL-1000, BioWhittaker), showing that recombinant histone preparations were essentially endotoxin-free (less than 30 ng endotoxin per mg of recombinant protein).

### Immunization Strategies

BALB/c mice were immunized three times, with a two-week interval between each immunization. Animals received three intramuscular (i.m.) injections of a DNA (100 µg) cocktail containing 25 µg of each recombinant plasmid (pcDNA3-LiH2A, pcDNA3-LiH2B, pcDNA3-LiH3 and pcDNA3-LiH4) in a total volume of 50 µl PBS. Control mice were immunized (i.m.) with 100 µg of WT pcDNA3. Alternatively, mice received two inoculations (i.m.) of the recombinant DNA cocktail followed by a third (s.c.) inoculation of a recombinant protein (20 µg) cocktail containing 5 µg of each recombinant protein (H2A, H2B, H3 and H4) and 25 µg of each CpG ODN (5′- tcagcgttga-3′ and 5′-gctagcgttagcgt-3′) (E-OLIGOS). Control mice received two immunizations (i.m.) with WT pcDNA3 followed by a third inoculation (s.c.) of saline+CpG. DNA immunizations were performed in the left quadriceps and protein immunizations were performed in the left footpad.

### Cytokine Detection in Immunized Mice

BALB/c mice were immunized as described above. Two weeks after the last immunization, mice were euthanized and single-cell suspensions of draining lymph nodes (dLN) were prepared aseptically. Briefly, dLN were homogenized in RPMI 1640 and cells were resuspended in RPMI supplemented with 2 mM L-glutamine, 100 U/mL penicillin, 100 µg/mL streptomycin, 10% FCS (all from Invitrogen) and 0.05 M β-mercaptoethanol. Cell suspensions were stimulated for 48 h with 5 µg/mL Concanavalin A (Amersham Biosciences) or 3 µg/mL of each of the following recombinant proteins: LiH2A, LiH2B, LiH3 and LiH4. Culture supernatants were harvested and cytokine concentrations were assayed using a Th1/Th2 cytokine Cytometric Bead Array (BD Biosciences), which detects murine IFN-γ, IL-4 and TNF-α according to the manufacturer’s instructions. The data were acquired and analyzed using a FACSort flow Cytometer (BD Immunocytometry) and CBA Analysis Software (Becton-Dickinson).

### Sand Flies and Preparation of SGS

*Lutzomyia intermedia* salivary glands were obtained as previously described [29]. Salivary gland from adult female flies were dissected and transferred to 10 or 20 µl Hepes, 10 mM pH 7.0 NaCl 0.15 in 1.5 polypropilene vials, usually in groups of 20 pairs of glands in 20 µl of Hepes saline. Salivary glands were kept at −75°C until needed, when they were disrupted by sonication using a Branson Sonifier 450 homogenizer (Branson, Danbury, CT). Salivary gland sonicate (SGS) was centrifuged at 10,000×g for 4 min and the supernatants were used in the experiments. The level of lipopolysaccharide (LPS) contamination of SGS preparations was determined using a commercially available LAL chromogenic kit (QCL-1000; Lonza Biologics). LPS concentration was <0.1 ng/ml.

### Parasite Culture, Intradermal Inoculation and Lesion Measurement

*L. braziliensis* promastigotes (strain MHOM/BR/01/BA788) [30] were grown in Schneider medium (Sigma), supplemented with 100 U/ml of penicillin, 100 µg/ml of streptomycin and 10% heat-inactivated fetal calf serum (Invitrogen). Two weeks after the last immunization, mice were inoculated with *L. braziliensis*, as described previously [30]. Challenges consisted of inoculation of stationary-phase promastigotes (10^5^ parasites)+SGS (1 µg, equivalent to 1 pair of salivary glands), in 10 µl of saline. Lesion size was monitored weekly for 10 weeks, using a digital caliper (Thomas Scientific).

### Parasite Load Estimate

Parasite load was determined using a quantitative limiting dilution assay as described previously [31]. Animals were euthanized five weeks post infection. Infected ears and draining lymph nodes (dLNs) were aseptically excised and homogenized in Schneider medium (Sigma). The homogenates were serially diluted in Schneider medium supplemented with 100 U/ml of penicillin, 100 µg/ml of streptomycin, 10% heat-inactivated fetal calf serum (all from Invitrogen) and seeded into 96-well plates containing biphasic blood agar (Novy-Nicolle-McNeal) medium. The number of viable parasites was determined from the highest to lowest dilution at which promastigotes could be grown after one week of incubation at 25°C.

### Histology

BALB/c mice were immunized and challenged as described above. Five weeks post challenge, animals were euthanized and ears were removed and fixed in 10% formaldehyde. Following fixation, tissues were processed, embedded in paraffin and 5 µm sections were stained with hematoxylin and eosin (H & E) and analyzed by light microscopy.

### RNA Isolation and Real-time PCR

Five weeks following infection with *L. braziliensis*+SGS, mice were euthanized and infected ears were excised and mechanically lysed with ceramic beads in a MagNALyzer® instrument (Roche Molecular Systems) according to manufacturer’s instructions. Total RNA was extracted from the resulting tissue lysates using the RNeasy Protect Mini Kit (Qiagen) according to the manufacturer’s instructions. RNA was eluted in 20 µl water and used for cDNA synthesis. Real-time PCR was performed on the ABI Prism 7500 (Applied Biosystems). Thermal cycle conditions consisted of a two-minute initial incubation at 50°C followed by a 10 minute denaturation at 95°C and 50 cycles at 95°C for 15 seconds and 60°C for one minute each. Each sample and the negative control were analyzed in triplicate for each run. The comparative method was used to analyze gene expression. Cytokine cycle threshold (C_t_) values were normalized to GAPDH expression as determined by the equation ΔC_t_ = C_t (cytokine)_ – C_t (GAPDH)_. Fold change was determined by 2^–ΔΔCt^, where ΔΔC_t_ = ΔC_t (experimental)_ – ΔC_t (control)._ [32]. Primers employed herein are described elsewhere [33].

### Intracellular Cytokine Detection by Flow Cytometry

Reagents for staining cell surface markers and intracellular cytokines were purchased from BD Biosciences. Measurement of in vitro cytokine production was performed as described [30]. Briefly, animals were euthanized five weeks post infection. dLNs were aseptically excised and homogenized in RPMI supplemented with 100 U/ml of penicillin, 100 µg/ml of streptomycin, 10% heat-inactivated fetal calf serum (all from Invitrogen). Cells were then activated using 5 µg/ml Concanavalin A (Amersham Biosciences) and incubated with 10 µg/ml Brefeldin A (Sigma). Cells were blocked with anti-Fc receptor antibody (2.4G2) and were double stained with anti-mouse surface CD4 (L3T4) and CD8 (53–6.7) conjugated to FITC and Cy-Chrome, respectively. For intracellular staining of cytokines, cells were permeabilized using Cytofix/Cytoperm and were incubated with the following anti-cytokine antibodies conjugated to PE: IFN-γ (XMG1.2), IL-4 (BVD4-1D11) or IL-10 (JES5-16E3). The isotype controls used were rat IgG2b (A95-1) and rat IgG2a (R35–95). Data were collected and analyzed using CELLQuest software and a FACSort flow cytometer (Becton Dickinson Immunocytometry System).

### Statistical Analysis

The data are presented as the mean ± SEM. Comparisons between four groups (DNA, DNA/Protein and controls) were performed by Kruskal-Wallis (non-parametric test) followed by Dunn’s multiple comparisons test. Comparisons between two groups (DNA vs. control or DNA/Protein vs. control) were performed by Mann-Whitney (non-parametric t-test) using GraphPad version 6.0a (Prism) and P-values <0.05 were considered significant. To evaluate disease burden in mice, ear thickness of mice following challenge was recorded weekly for each individual mouse. The course of disease for experimental and control mice was plotted individually. The Area under the curve (AUC) obtained for each mouse immunized with antigen versus AUC obtained for each control mouse was analyzed by Mann-Whitney (non-parametric t-test).

## Results

### Immunization with Nucleosomal Histones Prevents Lesion Development in Mice Infected by *L. braziliensis* Plus Sand Fly Saliva

**Figure 1:**
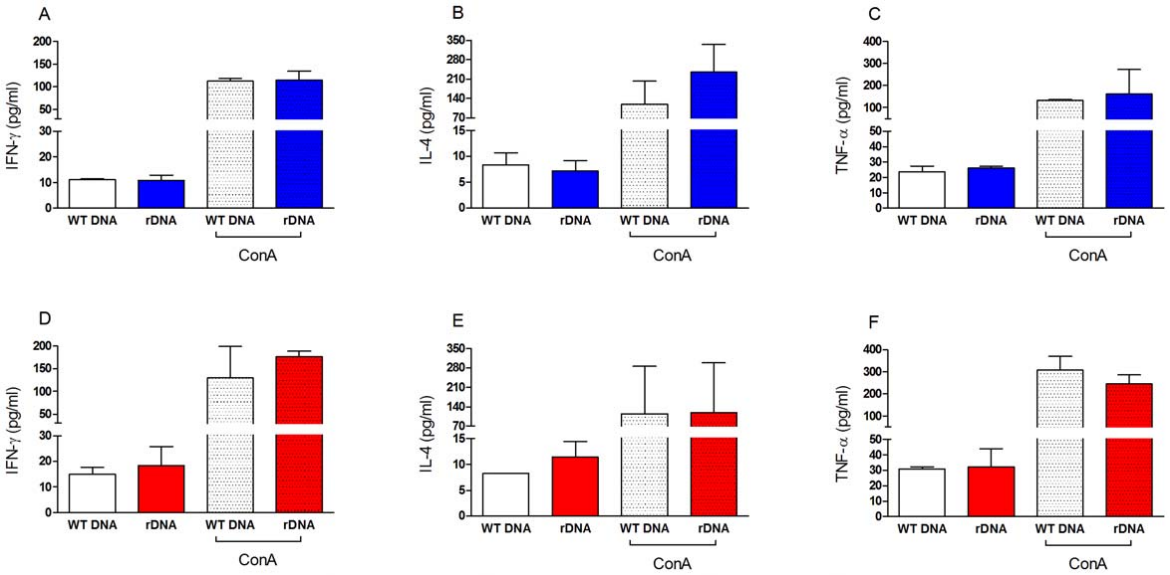
Antigen specific cytokine production following immunization with nucleosomal histones. BALB/c mice (5 per group) were immunized with wild type DNA (WT DNA) or with recombinant DNA coding for nucleosomal histones (rDNA) (A–C). Alternatively, BALB/c mice (5 per group) were immunized with wild type DNA followed by CpG (WT DNA+CpG), or with recombinant DNA followed by recombinant nucleosomal histones+CpG (rDNA/rProtein+CpG) (D–F). Two weeks after the last immunization, dLNs were collected and cells were re-stimulated with the recombinant proteins or with Concanavalin A (Con A). Antigen specific cytokine production in culture supernatants was determined by flow cytometry, using a Th1–Th2 Cytometric Bead Array. Data are presented as the mean+SEM and are from two independent experiments.

**Figure 2:**
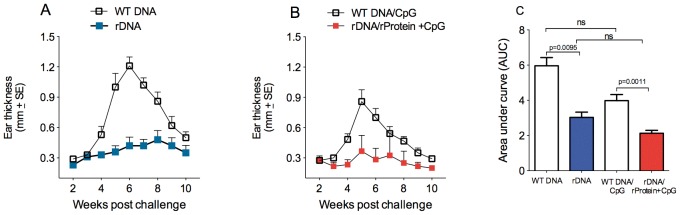
Lesion development in mice immunized with nucleosomal histones following infection with *L. braziliensis* plus sand fly saliva. BALB/c mice (5 per group) were immunized with WT DNA or with rDNA (A). Alternatively, BALB/c mice (5 per group) were immunized with WT DNA+CpG or with rDNA/rProtein+CpG (B). Two weeks after the last immunization, mice were challenged with *L. braziliensis*+sand fly saliva. The course of lesion development was monitored weekly and bars represent the means and standard errors from two independent experiments. The areas contained underneath the curves obtained in (A) and in (B) for each individual mouse from experimental and control groups were compared (C). Data are presented as the mean+SEM.

We first analyzed the immune response induced upon immunization with a plasmid DNA cocktail encoding histones H2A, H2B, H3 and H4. Mice inoculated with the recombinant DNA cocktail (rDNA) did not show a significant increase in IFN-γ (Fig. 1A), IL-4 (Fig. 1B) or TNF-α (Fig. 1C) when compared with mice immunized with WT DNA. Similar results were observed regarding antigen specific cytokine production in mice immunized with the combination of plasmid DNA followed by recombinant proteins (rDNA/rProtein+CpG) vs. control (WT DNA/CpG) (Fig. 1D–F). Two weeks after the last immunization, mice were challenged in the dermis of the ear by co-inoculation of *L. braziliensis* plus sand fly (*L. intermedia)* saliva, in order to mimic the natural context of infection by *Leishmania* parasites. Following challenge, lesion development was monitored for ten weeks. Mice immunized with rDNA did not develop disease, as shown by maintenance of ear thickness close to baseline levels (Fig. 2A), indicating disease prevention. Control mice (immunized with WT DNA) developed lesions that peaked at five weeks after infection (Fig. 2A), characteristic of the inoculation of *L. braziliensis* into the ear dermis of BALB/c mice [30]. At this time point, ear thickness reached a maximum of 1.2 mm. Mice inoculated with rDNA/rProtein+CpG (Fig. 2B) also did not develop lesions whereas controls immunized with WT DNA/CpG behaved as mice inoculated with WT DNA (Fig. 2A). Importantly, disease development, as determined by the area under the curves (AUCs) shown in Fig. 2A and Fig. 2B (see Materials and Methods), was significantly inhibited in mice immunized with rDNA or with rDNA/rProtein+CpG, when compared with controls (Fig. 2C). The AUCs of immunized mice (either rDNA only or rDNA/rProtein+CpG) and of control mice (WT DNA only or WT DNA/CpG) were similar. This suggests that immunization with *L. infantum chagasi* histones inhibit cutaneous leishmaniasis caused by *L. braziliensis*.

**Figure 3:**
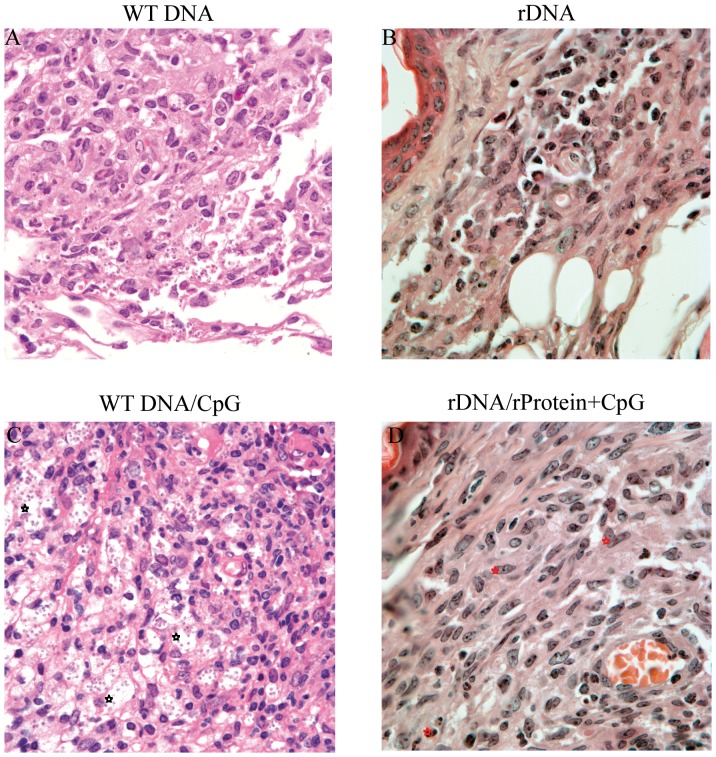
Histological aspects of ear lesions in mice immunized with nucleosomal histones and challenged with *L. braziliensis* plus sand fly saliva. BALB/c mice (five mice per group) were immunized with WT DNA (A) or with rDNA (B). Alternatively, mice were immunized with WT DNA/CpG (C) or with rDNA/rProtein+CpG (D). Two weeks after the last inoculation, mice were challenged with *L. braziliensis*+sand fly saliva. Ears were removed at 5 weeks post infection and stained with hematoxylin and eosin. Panels represent 100×magnification. (C) Dark symbols indicate infected macrophages displaying a foamy aspect. (D) Red symbols indicate plasmocytes and dashed arrows indicate eosinophils.

We also analyzed ear sections, obtained five weeks after co-inoculation with *L. braziliensis* plus sand fly (*L. intermedia)* saliva. As shown in Fig. 3A, mice immunized with WT DNA developed an intense inflammatory infiltrate with an accumulation of parasite-infected macrophages. In mice immunized with WT DNA, infected cells were abundant (Fig. 3A), differently from mice immunized with rDNA (Fig. 3B). In tissue sections from mice immunized with WT DNA/CpG, we observed a dense and widespread inflammatory infiltrate containing infected macrophages displaying a foamy aspect (Fig. 3C). In rDNA/rProtein+CpG-inoculated mice, amastigotes were not detected and the inflammatory infiltrate was characterized by the presence of rare eosinophils, plasmocytes and epithelioid macrophages (Fig. 3D).

### Parasite Load and Cytokine Expression Profile at the Infection Site

**Figure 4:**
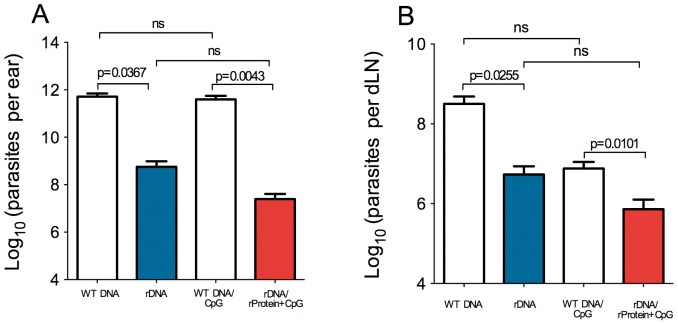
Parasite load following immunization with nucleosomal histones and challenge with *L. braziliensis* plus sand fly saliva. BALB/c mice (5 per group) were immunized with WT DNA or with rDNA. Alternatively, BALB/c mice (5 per group) were immunized with rDNA/rProtein+CpG. Two weeks after the last immunization, mice were challenged with *L. braziliensis*+sand fly saliva. Ear (A) and draining lymph node (B) parasite loads were determined five weeks post infection via a limiting dilution assay. Data are presented as the mean+SEM and are from two independent experiments.

Given the significant inhibition of lesion development following immunization with plasmid rDNA (Fig. 2A and C) or with rDNA/rProtein+CpG (Fig. 2B and C), we also investigated the parasite load. At five weeks post parasite inoculation, when ear thickness was at its peak (Fig. 2A and B), mice immunized with rDNA displayed a significantly lower parasite load at the infection site (Fig. 4A) and at the draining lymph node (Fig. 4B). Comparable results were observed with rDNA/rProtein+CpG-immunized mice (Fig. 4A and B), in terms of inhibition in parasite replication. The parasite load detected in the ear of mice immunized with histones (rDNA vs. rDNA/rProtein+CpG) or in control animals (WT DNA vs. WT DNA/CpG) were not significantly different (Fig. 4A and B).

Therefore, immunization with *L. infantum chagasi* nucleosomal histones modified the course of infection in mice challenged with *L. braziliensis*.

**Figure 5:**
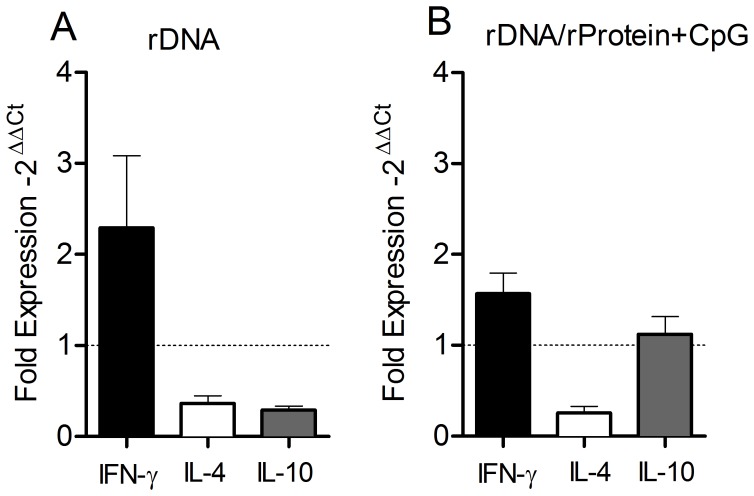
Cytokine expression in the ear dermis following immunization with nucleosomal histones and challenge with *L. braziliensis* plus sand fly saliva. BALB/c mice (5 per group) were immunized with rDNA (A) or with rDNA/rProtein+CpG (B). Two weeks after the last immunization, mice were challenged with *L. braziliensis*+sand fly saliva. Relative quantification of IFN-γ, IL-4 and IL-10 at the infection site was carried out five weeks after infection. Cytokine cycle threshold (C_t_) values were normalized to GAPDH expression (housekeeping) as determined by ΔC_t_ = C_t (cytokine)_ – C_t (GAPDH)_. Fold change was determined by real-time PCR, using the 2^–ΔΔCt^ method, where ΔΔC_t_ = ΔC_t (experimental)_ – ΔC_t (control)_ (see Materials and Methods). Data (mean+SEM) are presented as fold increase in gene expression of immunized mice over control mice and are from two independent experiments.

Based on these results, we analyzed cytokine expression at the infection site. Total RNA was obtained from ear sections five weeks after parasite inoculation, when ear thickness was at its peak in control mice (Fig. 2) and the parasite load was significantly different between immunized versus control groups (Fig. 4). RNA was subjected to real-time PCR analysis, and cytokine gene expression was normalized to GAPDH (housekeeping gene), as described in the Materials and Methods. Ears from rDNA-immunized mice showed a two-fold up-regulation in IFN-γ expression in comparison with control mice (Fig. 5A) whereas the expression of IL-4 and IL-10 were down-regulated (Fig. 5A). Mice immunized with rDNA/rProtein+CpG also showed up-regulation of IFN-γ expression (Fig. 5B). These results indicate the predominance of a Th1-polarized response and can be correlated with the decreased disease burden (Fig. 2) and the lower parasite load observed at the infection site (Fig. 4) of mice immunized with nucleosomal histones.

### Cellular Immune Response in dLNs

**Figure 6:**
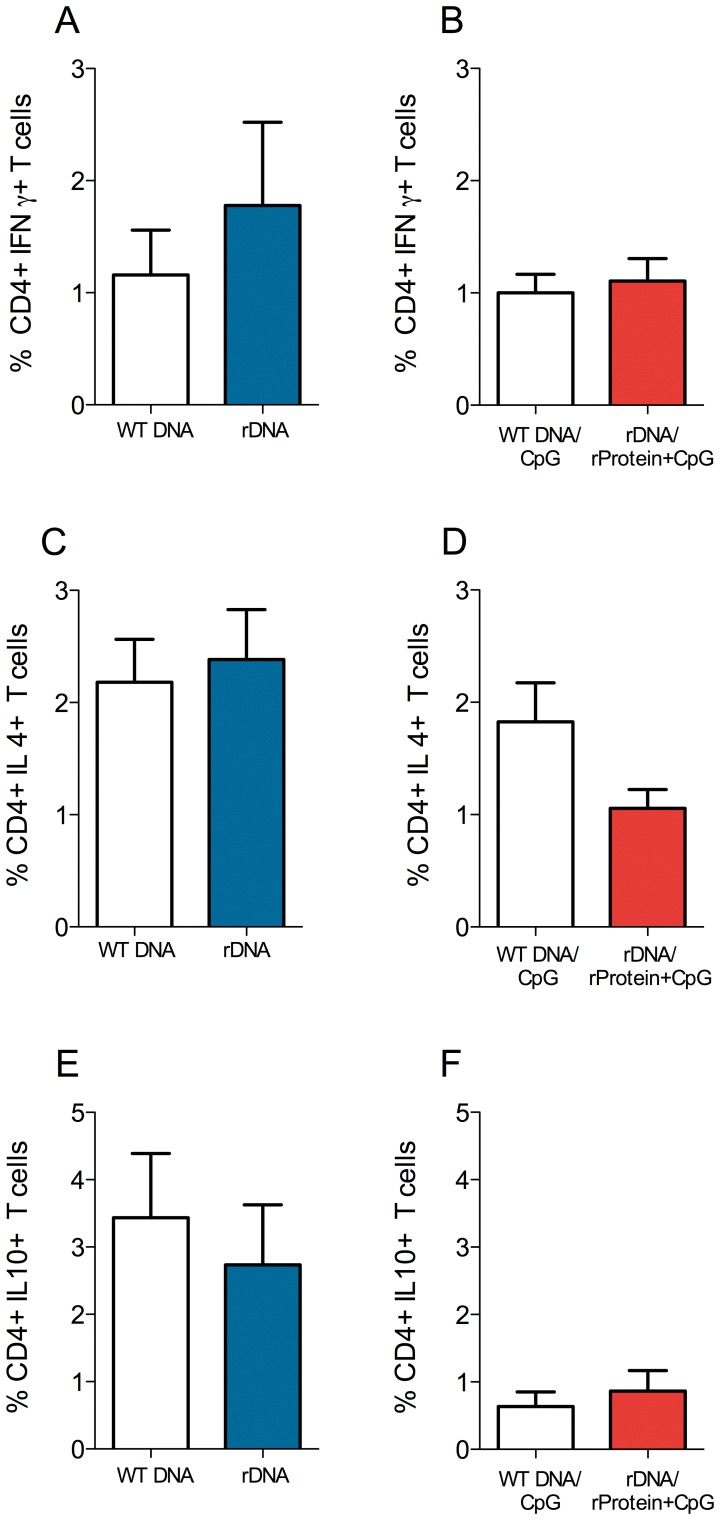
Intracellular cytokine production by CD4+ cells from mice immunized with nucleosomal histones and challenged with *L. braziliensis* plus sand fly saliva. BALB/c mice (5 per group) were immunized as described. Two weeks after the last immunization, mice were challenged with *L. braziliensis*+sand fly saliva. Five weeks later, draining lymph nodes were pooled and cells were preincubated with Brefeldin A for four hours before staining. Data represent the frequency of CD4+ cells positive for IFN-γ (A, B), IL-4 (C, D) and IL-10 (D, E) with signals for the particular cytokine that were greater than the background signals established using isotype controls. Data are presented as the mean+SEM and are from two independent experiments.

**Figure 7:**
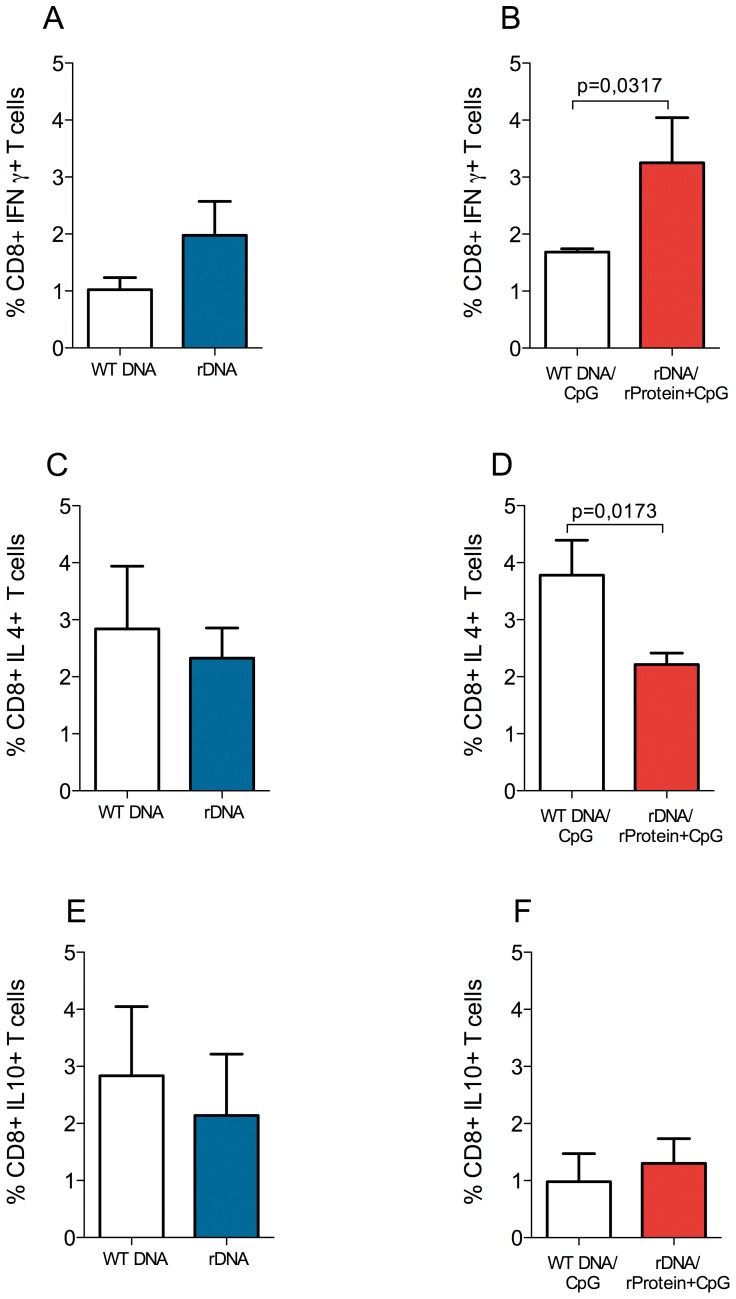
Intracellular cytokine production by CD8+ cells from mice immunized with nucleosomal histones and challenged with *L. braziliensis* plus sand fly saliva. BALB/c mice (5 per group) were immunized as described. Two weeks after the last immunization, mice were challenged with *L. braziliensis*+sand fly saliva. Five weeks later, draining lymph nodes were pooled and cells were preincubated with Brefeldin A for four hours before staining. Data represent the frequency of CD8+ cells positive for IFN-γ (A, B), IL-4 (C, D) and IL-10 (D, E) with signals for the particular cytokine that were greater than the background signals established using isotype controls. Data are presented as the mean+SEM and are from two independent experiments.

Based on the lower parasite load observed in the dLNs of immunized mice (Fig. 2), we also evaluated the presence of cytokine-secreting cells therein. Five weeks after infection, cells from lymph nodes draining the infection site were re-stimulated in vitro with recombinant histones and the frequency of cytokine-secreting cells was determined by flow cytometry (Figure S1). The percentage of CD4+ IFN-γ-secreting cells was similar in control vs. rDNA-immunized mice (Fig. 6A) and in controls vs. rDNA/rProtein+CpG-immunized mice (Fig. 6B). Also, we did not detect significant differences in the percentage of CD4+ IL-4+-secreting cells in (Fig. 6C–D) or in the frequency of CD4+IL-10+-secreting cells (Fig. 6E–F), when comparing the two immunization strategies. The percentage of CD8+ IFN-γ-secreting cells was similar in control vs. rDNA-immunized mice (Fig. 7A) but was significantly higher in mice immunized with rDNA/rProtein+CpG when compared with controls (Fig. 7B). The frequency of CD8+IL-4+-secreting cells was also similar in control vs. rDNA-immunized mice (Fig. 7C) but was significantly lower in mice immunized with rDNA/rProtein+CpG when compared with controls (Fig. 7D). As seen with CD4+ cells, the frequency of CD8+IL-10+-secreting cells did not differ significantly in control vs. immunized mice (Fig. 7E and F).

## Discussion

In the present work, we evaluated the comparative vaccine potential of rDNA and rDNA/rProtein+CpG using *L. infantum chagasi* histones H2A, H2B, H3 and H4 in the context of eliciting immunity against *L. braziliensis.* The results reported here suggest that both strategies were able to prevent lesion development in an experimental model of New World cutaneous leishmaniasis.

Previous studies using *L. infantum chagasi* nucleosomal histones have shown the induction of a Th1-biased response, with significant levels of protection against *L. major* infection in mice [19], [20], [21]. To evaluate efficacy in an experimental model of New World Cutaneous Leishmaniasis, we employed two immunization strategies: rDNA and rDNA/rProtein+CpG. The prime-boost strategy (rDNA/rProtein+CpG) aims at augmenting immune responses induced by rDNA vaccination alone and it has been successfully employed previously in leishmaniasis [34], [35], [36], [37]. Here, recombinant proteins were formulated with CpG, given its ability to stimulate macrophages and dendritic cells (DCs) to synthesize cytokines, up-regulate the expression of co-stimulatory molecules and to enhance the cross-presentation properties of DCs [38], [39]. In the present study, immunization with rDNA alone or with rDNA followed by rProtein+CpG did not elicit a strong immune response, as shown by the lack of a significant increase in cytokine production in immunized mice. Herein, cell cultures were simultaneously stimulated with the recombinant proteins corresponding to histones H2A, H2B, H3 and H4 whereas in the study by Iborra et al., cells from mice immunized with nucleosomal histones were stimulated with each recombinant protein separately and the authors were able to detect a significant increase in IFN-γ secretion [21].

Despite the fact that we did not detect a robust pre-challenge immune response, both immunization strategies were able to significantly inhibit lesion development upon intradermal inoculation of *L. braziliensis,* in the presence of *L. intermedia* sand fly saliva. Although mice were challenged with a high inoculum (10^5^ stationary-phase promastigote forms), significant protection was achieved as shown by lack of lesion development and by the significant reduction (over 2 log) in parasite load observed at the infection site in mice vaccinated with either rDNA or rDNA/rProtein+CpG. This level of protection is comparable to vaccination studies published employing nucleosomal histones and *L. major* infection [40]. Regarding sand fly saliva, it contains pharmacologically active molecules that promote adequate blood feeding and that may contribute to establishment of infection by *Leishmania* (rev. in [41]). Among the salivary components characterized to date are maxadilan [42], that modulates the inflammatory response by inhibiting cytokines such as TNF-*α*; hyaluronidase, that helps the diffusion of other pharmacological substances through the skin matrix [43] and a 5-nucleotidase, that exerts a vasodilator and anti-platelet aggregation role by converting AMP to adenosine [44]. In the case of *L. braziliensis*, co-inoculation of salivary gland sonicate (SGS) and parasites led to a significant exacerbation of both lesion size and parasite load [45], [46], [47]. We later showed that immunization with *L. intermedia* SGS altered the course of experimental infection with *L. braziliensis* [29] and stimulation of immune mice with a combination of SGS and *L. braziliensis* led to a decreased CXCL10 expression and increased IL-10 expression [33]. These results suggest that *L. intermedia* saliva exerts important immunomodulatory activities and, therefore, we judged important to include salivary components to the challenge inoculum. Other studies also employed salivary gland sonicate to “mimic” the effects of sand fly saliva in the context of vaccination [48], [49], [50], [51], [52]. More recently, Peters et al. showed that vaccination with autoclaved *L. major* antigen+CpG confers protection against a needle inoculation of parasites but not against challenge with infected sand flies [53] whereas KSAC, a polyprotein vaccine candidate [54], conferred protection against the bite of *L. major-*infected sand flies [55]. To date, a colony of *L. intermedia* sand flies is not available, hampering the possibility of using infected sand flies in challenge experiments. In this sense, we believe that addition of SGS at the time of challenge partially addresses this limitation and allows us to evaluate the effects of salivary molecules in the context of vaccination.

Inhibition of lesion development in mice immunized with nucleosomal histones (rDNA or rDNA/rProtein+CpG) was accompanied by a significant decrease in parasite load at the infection site. These results may be related to a significant up-regulation of the expression of IFN-γ, as seen in infected ears five weeks after infection. Macrophages control *Leishmania* infection by inducing reactive oxygen species when activated by IFN-γ [56]. Therefore, we can speculate that IFN-γ-secreting cells may have migrated to the infection site, promoting macrophage activation and parasite killing. Additionally, Iborra et al. showed 1.22% of CD4+ IFN-γ+ and 1.02% of CD8+ IFN-γ+ T cells in mice challenged with *L. major* [21] whereas we detected 1.7% CD4+IFN-γ+ (Figure 6) and 1.9% CD8+ IFN-γ+ cells (Figure 7), in mice immunized with rDNA and challenged with *L. braziliensis*. We believe these results are comparable in terms of the immune response detected after challenge and indicate that the protective capacity of nucleosomal histones is associated with an expansion of IFN-γ-expressing cells.

Chenik et al. documented the participation of the carboxy-terminal portion of the H2B histone in the activation of Treg cells [18]. The presence IL-10 secreting cells, with a regulatory phenotype, may have modulated potentially harmful effects associated with the development of a Th1-immune response [57], leading to the control of pathology at the infection site of mice immunized with nucleossomal histones. Indeed, the presence of Tregs has been associated with healing of experimental infection with *L. braziliensis* [58]. On the other hand, control mice that did not receive nucleosomal histomes did not develop a Th1-immune response. In this case, the potential presence of a regulatory response could have contributed with parasite replication instead of a control in pathology.

During the past several decades, extensive efforts have been made to develop an effective *Leishmania* vaccine [3], [22]. The majority of studies have been conducted with *L. major* [1]. *L. braziliensis*, which is distinguished from other leishmaniasis by its chronicity, latency and tendency to metastasize in the human host [59], has been largely neglected in the context of vaccine development. Moreover, candidate antigens such as the receptor for activated C kinase protein (LACK), thiol-specific antioxidant (TSA), *Leishmania* elongation and initiation factor (LeIF) and *L. major* stress-inducible protein 1 (LmST1), all of which induced protection against *L. major*, failed to prevent *L. braziliensis* infection [60]. These findings highlight the need for continued investigation into molecules able to confer protection against this particular species. In conclusion, we believe our results represent an important contribution to understanding leishmaniasis as they extend the cross-protective effect of nucleosomal histones from *L. infantum chagasi* to a model of New World cutaneous leishmaniasis caused by *L. braziliensis*.

## Supporting Information

Figure S1 **Cytokine expression in CD4+ and in CD8+ cells in mice immunized with nucleossomal histones, following challenge with** ***L. braziliensis*** **plus sand fly saliva.** BALB/c mice were immunized with DNA coding for nucleosomal histones and two weeks after the last immunization, mice were infected in the dermis of the ear with 10^5^ *L. braziliensis+ sand fly saliva,* as described in Materials and Methods. Gates depict CD4+and CD8+ T lymphocytes present in the draining lymph node (dLN). The presence of IFN-γ^+^, IL-4^+^ and IL-10^+^ T cells was determined by flow cytometry in the gated populations. Data shown are representative dot plots for IFN-γ^+,^ IL-4^+^ and IL-10 labeling.
(TIF)
