# Supplementary figures and images for: Vaccination with L. infantum chagasi Nucleosomal Histones Confers Protection against New World Cutaneous Leishmaniasis Caused by Leishmania braziliensis
Source: PLoS One. 2012 Dec 20;7(12):e52296. doi: 10.1371/journal.pone.0052296 (PMC3527524; doi:10.1371/journal.pone.0052296)

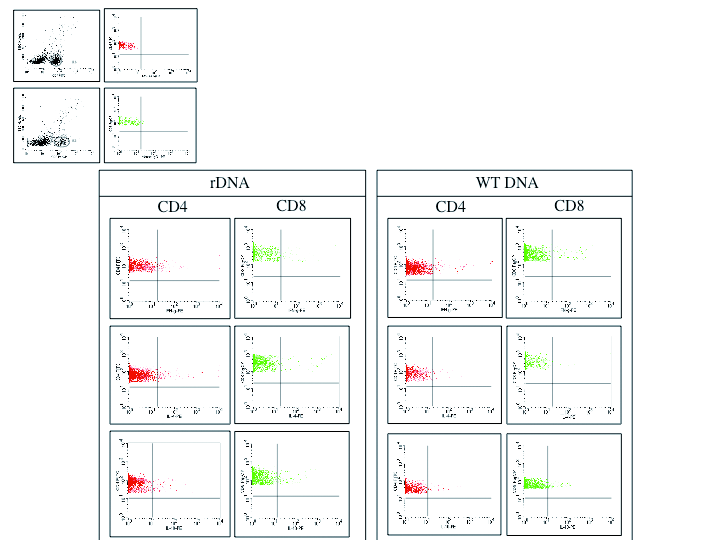

Supplement: Figure S1 — Cytokine expression in CD4+ and in CD8+ cells in mice immunized with nucleossomal histones, following challenge with L. braziliensis plus sand fly saliva. BALB/c mice were immunized with DNA coding for nucleosomal histones and two weeks after the last immunization, mice were infected in the dermis of the ear with 105 L. braziliensis+ sand fly saliva, as described in Materials and Methods. Gates depict CD4+and CD8+ T lymphocytes present in the draining lymph node (dLN). The presence of IFN-γ+, IL-4+ and IL-10+ T cells was determined by flow cytometry in the gated populations. Data shown are representative dot plots for IFN-γ+, IL-4+ and IL-10 labeling. (TIF) [file pone.0052296.s001.tif]
